# Endogenous DNA Damage and Its Role in Human Disease

**DOI:** 10.1021/acs.chemrestox.5c00539

**Published:** 2026-02-18

**Authors:** Eva-Maria Bryan, Clare Lauderback

**Affiliations:** Department of Chemistry and Biochemistry, Institute for Advanced Biomedical Research, 3298 George Mason University, Manassas, Virginia 22030, United States

**Keywords:** Endogenous DNA Damage, DNA Repair Pathways, Secondary DNA Structures, Metabolic Dysfunction, Genomic Instability

## Abstract

Endogenous DNA damage from metabolism and genome dynamics drives mutation, instability, and disease. New insights from ACS Fall 2025 reveal chemical diversity, metabolic drivers, and repair limits that shape cancer risk and biomarker development.

## Graphical abstract

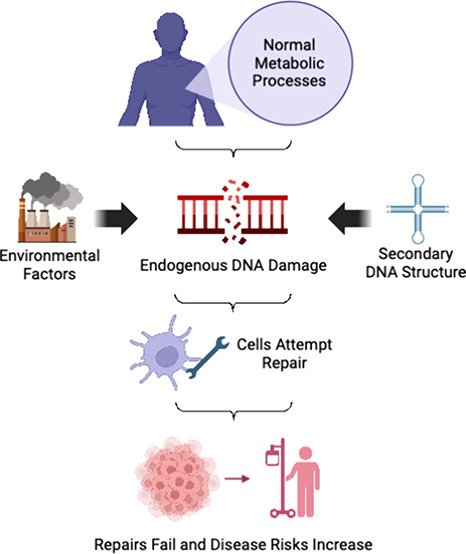

Endogenous DNA (Deoxyribonucleic acid) damage results from normal metabolic processes such as lipid peroxidation, spontaneous base deamination, and structural transitions within the genome. This generated a diverse landscape of DNA lesions that challenge cellular homeostasis. Recent work presented at the ACS Fall 2025 symposium “Endogenous DNA Damage and Its Role in Human Disease” highlights how these lesions cause mutagenesis, genomic instability, and disease risk.^1^ The presenters in this symposium gave valuable insights and underscored the complexity and biomedical importance of endogenous DNA damage.
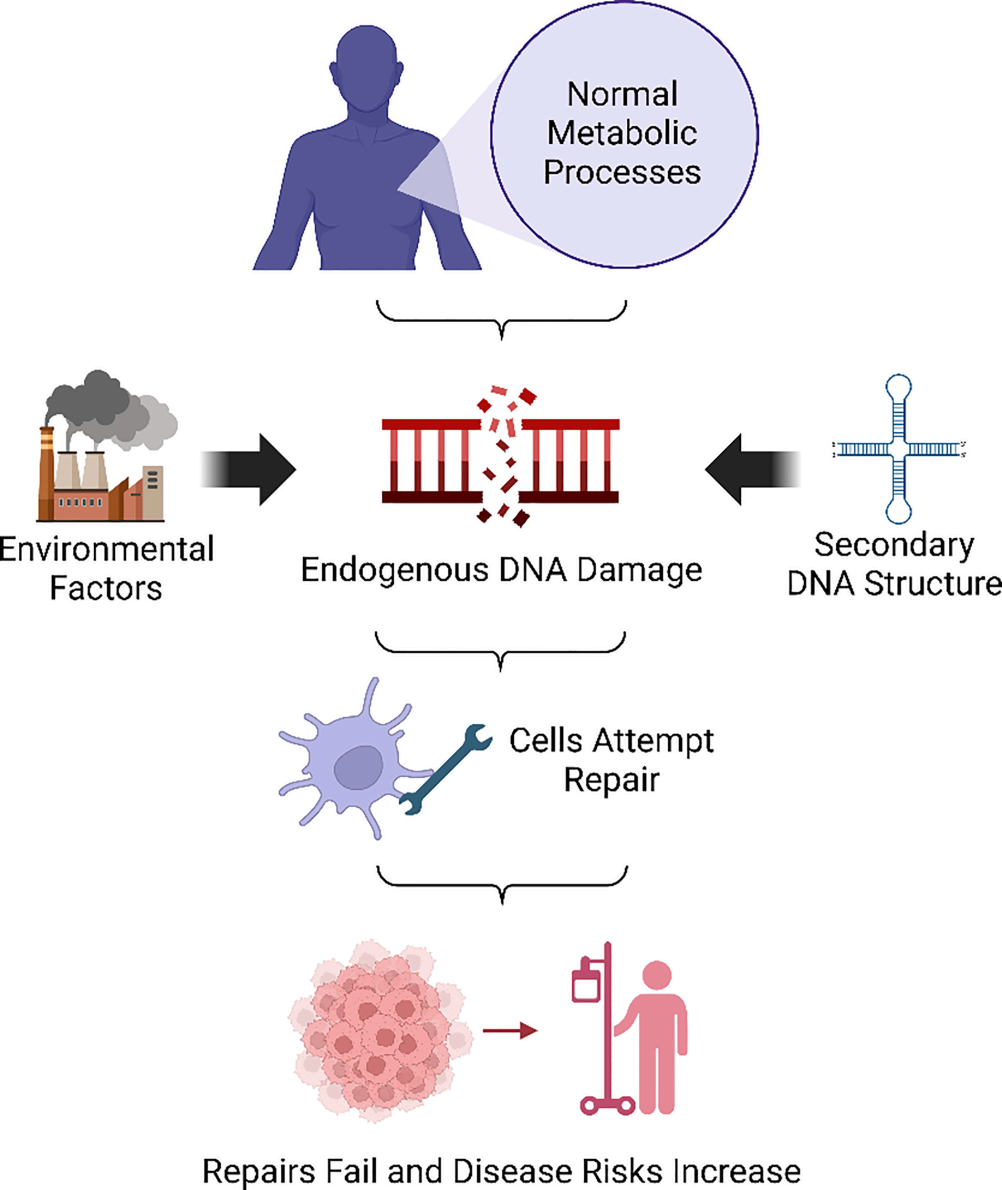


A central theme of the symposium was the chemical diversity of endogenous DNA lesions and the unexpected complexities that arise when cells attempt to repair them. The first speaker, Dr. Deyu Li, opened this discussion with his presentation titled “Chemistry and biology of etheno DNA adducts repair,” outlining new mechanistic insights into how these lesions form, persist and repair. They reported a novel type of DNA interstrand cross-link (ICL) produced as a side product during the attempted repair etheno adducts by the human Fe­(II)/α-ketoglutarate-dependent enzyme ALKBH2. Among the etheno adducts examined, ethenocytosine produced the highest ICL yield, prompting deeper investigation into its formation and repair. Their recent findings reveal that ABH2, ABH3, and FTO can dealkylate etheno-5-methylcytosine, whereas BER shows no activity—highlighting why this lesion may remain unrepaired and contribute to genetics and epigenetics modulations.^2^ Building directly on this theme of distinguishing lesion origins and repair outcomes, Dr. Natalia Tretyakova presented her work on “Isotope labeling to distinguish between endogenous and exogenous DNA adducts.” Her research findings show that more than 90% of N-7-(2,3,4-trihydroxybutyl)­guanine (THBG) and a substantial fraction of 1,2-dihydroxy-3,4-epoxybutane-derived mercapturic acid (DHBMA) (butadiene-derived adducts) arise endogenously, even in the absence of butadiene (BD) exposure, underscoring how metabolic processes can overshadow environmental contributions. Nutrient-labeling studies further showed that erythrose, erythritol, and glucose do not influence THBG formation. Together, these results clarify the metabolic origins of BD-related adducts and refine biomarker interpretation. Collectively, these studies reinforce that endogenous DNA adducts are not static byproducts of normal metabolism but dynamic chemical species whose formation and repair can introduce additional layers of genomic instability—emphasizing the need for more precise tools to track their origins, reactivity, and biological consequences.^3^

Beyond chemically induced lesions, the symposium also highlighted structural features of the genome, such as unusual DNA structures like H and Z DNA, as inherent sources of mutagenesis in cancer genomes. In her presentation titled “Novel mechanisms of genetic instability in cancer,” Dr. Karen Vasquez presented compelling evidence that DNA secondary structures—such as H-DNA, Z-DNA, and cruciforms—are important endogenous sources of genomic instability with direct relevance to cancer development. Overall, Vasquez’s work underscores that non-B DNA structures are both functional genomic elements and intrinsic threats to genome integrity, positioning them as key contributors to endogenous DNA damage and cancer biology. These findings reveal that genomic instability can originate from the physical architecture of DNA itself, broadening the definition of endogenous damage to include shape-based mechanisms of mutation.^4^

While DNA secondary structures destabilize the genome through physical mechanisms, other talks focused on enzymatic sources of endogenous mutagenesis and how these interact with environmental exposures. Another critical insight from the symposium was that endogenous mutational processes, such as Apolipoprotein B mRNA-editing enzyme, catalytic polypeptide-like (APOBEC) cytosine deamination, can interact with environmental carcinogens to increase mutation rates far beyond what either process produces alone.^5^ In his presentation titled “Tobacco smoke carcinogens exacerbate APOBEC mutagenesis and carcinogenesis,” Dr. Reuben S. Harris examined the origins of cancer-associated mutations, emphasizing that distinct mutational signatures reflect specific sources of DNA damage. Computational analyses reveal that smokers show elevated APOBEC mutagenesis and didyma in both tumors and normal lung tissue, indicating that tobacco-derived DNA-adducting mutagens can amplify endogenous APOBEC-driven DNA damage, highlighting how mutational mechanisms can act synergistically during cancer initiation and promotion.^6^ This synergy shows that mutational pathways do not operate independently and that understanding disease risk requires examining how endogenous and exogenous mechanisms interact within the same biological environment.

Another layer of endogenous DNA damage comes not from nucleic acid chemistry or structure, but from metabolic byproducts that accumulate under disease conditions. The role of endogenous DNA damage in metabolism was emphasized by new evidence indicating that methylglyoxal-derived adducts play a part in the development of both diabetes and cancer. Dr. Sarah Shuck presented a compelling talk titled “Regulating methylglyoxal toxicity: shedding light on the diabetes-cancer connection,” where she explored how metabolic dysfunction, particularly obesity and diabetes, creates a biochemical environment that promotes prostate cancer progression. Methylglyoxal (MG), a highly reactive dicarbonyl, arises primarily as a byproduct of glycolysis through spontaneous triose phosphate breakdown, making its accumulation a characteristic feature of metabolic imbalance. She highlighted evidence that reduced Glyoxalase 1 (GL01) expression, especially in prostate cancer cells derived from African American men, leads to elevated MG-DNA adducts and increased mutation frequencies consistent with COSMIC Cysteine→Threonine signatures. Multiple repair pathways can detect MG lesions, but their loss amplifies mutagenesis. Shuck also presented emerging data suggesting that GLP-1 receptor agonists may reduce MG-adduct burden and improve tumor responses through metabolic and Nicotinamide Adenine Dinucleotide (NAD)-pathway restoration. By revealing how metabolic dysfunction increases DNA damage and influences disease susceptibility, this work identifies endogenous mutagenesis as an essential molecular link between chronic metabolic disorders and cancer risk.^7^

The symposium concluded by highlighting emerging technologies such as nanopore-based sequencing, which enable direct detection of endogenous DNA lesions and epigenetic modifications at single-nucleotide resolution. Dr. Gunner Boysen presented new work entitled “Studying the dynamic changes of DNA modifications and the adductome in single cells using nanopore sequencing,” applying these tools to track dynamic changes in DNA modifications and the cellular adductome. A recently developed nanopore-based DNA sequencing approach named ELIGOS (Epitranscriptional/(Epigenomical) Landscape Inferring from Glitches of ONT Signals) can detect and distinguish DNA base adducts, ribose-phosphate backbone modifications, epigenetic marks, exposure-derived adducts, and common endogenous lesions with single-nucleotide precision.^8,9^ These capabilities provide real-time insight into chemical alterations within individual DNA molecules, offering powerful new ways to connect specific DNA modifications to mutational outcomes, biomarkers, and potential therapeutic strategies.^8^ Together, these advances set the stage for future progress in understanding the origins, consequences, and biomedical significance of endogenous DNA damage.

## Abbreviations

DNA: Deoxyribonucleic Acid
LC-MS/MS: Liquid Chromatography-Tandem Mass Spectrometry
ICL: Interstrand Cross-link
ALKBH2: AlkB Homologue 2
ALKBH3: AlkB Homologue 3
FTO: Fat Mass and Obesity-Associated Protein
BER: Base Excision Repair
THBG: N-7-(2,3,4-Trihydroxybutyl)­guanine
DHBMA: 1,2-Dihydroxy-3,4-epoxybutane-derived Mercapturic Acid
BD: 1,3-Butadiene
H-DNA: H-form DNA
Z-DNA: Z-form DNA
APOBEC: Apolipoprotein B mRNA-Editing Enzyme, Catalytic Polypeptide-Like
MG: Methylglyoxal
GLO1: Glyoxalase 1
GLP-1: Glucagon-Like Peptide-1
NAD: Nicotinamide Adenine Dinucleotide
ELIGOS: Epitranscriptional/Epigenomical Landscape Inferring from Glitches of ONT Signals
ONT: Oxford Nanopore Technologies.
